# Research on Forming Limit Stress Diagram of Advanced High Strength Dual-Phase Steel Sheets

**DOI:** 10.3390/ma16134543

**Published:** 2023-06-23

**Authors:** Hongjian Cui, Di Li, Qiutao Fu, Zipeng Lu, Jiachuan Xu, Ning Jiang

**Affiliations:** 1 School of Transportation and Vehicle Engineering, Shandong University of Technology, Zibo 255000, China; 2 School of Automobile Engineering, Zibo Vocational Institute, Zibo 255300, China

**Keywords:** advanced high-strength steel, forming limit diagram, forming limit stress diagram, failure criterion

## Abstract

The Forming Limit Stress Diagram (FLSD) can accurately describe the forming process of high-strength steel. However, obtaining FLSD is relatively difficult. In order to predict fracture in advanced high-strength dual-phase (DP) steels, limit maximum and limit minimum principal strains of sheet were obtained through multiple sets of test and simulation. Two material parameters, strength coefficient *K* and hardening exponent *n* are introduced into the FLSD function which is established by the strain-stress transformation function. The function shows that the *k*-value determines the value of the maximum principal stress, while the *n*-value affects the curvature of the curve. Verification of correctness by testing and simulation to within 10% accuracy. This paper explores a new approach to FLSD research based on material properties, which can expand the application scope of FLSD.

## 1. Introduction

As excellent automotive lightweight material, Advanced high-strength steels (AHSS), especially advanced high-strength dual-phase (DP) steels are widely used in body structures with high-strength requirements. Usually, as with other metallic materials, the formability of AHSS sheet is assessed by forming limit stress diagram (FLD). FLD is represented by a curve in the principal strain coordinate system, which divides the state space of the two principal strains (all stress states from uniaxial tension to biaxial tension) in the sheet into two regions: a safety zone and a fracture zone.

However, although widely used, the current FLD is unsatisfactory to describe AHSS forming. Due to the local plastic deformation of the edge, the property of the AHSS sheet processed by the shear process has changed significantly. Their uneven hardening phenomenon of the shear edge area is more prominent, which greatly improves the probability of edge cracking of the material in the subsequent forming process. Wang K et al. [1] found that when the pre-strain is larger than the the limit strain which can be obtained under linear path, the FLD will become very unstable due to the path influence. Another typical problem that occurs during stamping of AHSS is the shear fracture [2,3]. Fu Q T et al. [4] investigated the strength coefficient *K*, hardening index *n*, and stress triaxiality associated with this shear fracture.

In order to avoid the springback in the forming process and improve the size precision of forming, the smaller bending radius is employed in designing of the mold, meanwhile increase the blank holder force during the forming as much as possible without creating fractures. With the decrease of the bending angle of the mold, part of the material close to the bending angle of the mold is easy to produce a shear fracture. There is no significant necking and almost no thinning at the shear fracture. Both of these phenomena are hard to predict in FLD. Numerous studies have found that FLD has disadvantages of strain-path dependent, which makes this method become ineffective in the analysis of complex forming process [5]. This feature makes it impossible to apply FLD to guide production in many cases.

It is necessary to develop a stress-based forming limit criterion independent of path in AHSS forming analysis. Kleemola H J and Pelkkikangas MT [6] first found that the limit strain is only determined by the stress state, and the stress value of its instability point is independent of the strain path. Therefore, they drew a curve with limit stress as the forming criterion, and analyzed alloy steel and other materials through the established curve. Forming Limit Stress Curve (FLSC), also known as Forming Limit Stress Diagram (FLSD) is one of stress-based, strain-path independent limiting criteria which can replace the FLD [7]. Arrieux R [8] calculates the axial and non-axial FLD of anisotropic materials through different strain paths and converts it to FLSD through flow rules. Analysis shows that FLSD can more effectively predict the necking start time than the traditional FLD. It is worth noting that a study by Sojodi S et al. [9] demonstrated that the FLSD is also affected by loading paths when the pre-strain increases to the level of the equivalent plastic strain under plane strain state. At present, the mechanism of FLSD formation is not fully understood and needs to be studied. Vadavadagi B H et al. [10] determined FLD and FLSD of IF and DQ steel sheets and found that higher hardening index *n* and plastic strain ratio *r* increase forming limit strains and stress.

It is difficult to obtain FLSD directly through testing. In existing studies, FLSD can be obtained by converting strain into stress according to the function, alternatively, directly in numerical simulation analysis. Huang T et al. [11] used GTN damage model parameters obtained by finite element reverse calibration to build simulation models to plot FLD and FLSD which was in good agreement with the experimental results. Yuan S et al. [12] transformed the experimentally obtained limit strain into limit stress by Hill’48 yield criterion and plotted the FLSD of low temperature alloy sheet.

So far, research on FLSD parameters of various metal materials is insufficient, which makes FLSD difficult to be used in engineering. Most previous studies in the field of FLSD have focused on a single steel grade, lacking a holistic analysis of a series of steel grades. The establishment of FLSD for DP steel of different grades can improve its application scope, meanwhile, it is helpful to optimize the simulation accuracy of stamping simulation.

Nakazima dome test is a common method for determining FLD. In this paper, Nakazima tests are carried out on four kinds of DP steel, DP590, DP780, DP980 and DP1180. The forming process of four kinds of DP steel was simulated and analyzed by using the user material subprogram of Abaqus Explicit. The FLD of DP steel was obtained by processing the maximum and minimum principal strain data obtained from test and simulation. According to the stress-strain transformation equation, the FLSC functions of four DP steels are obtained. The relationship between material properties and FLSD was analyzed. Finally, the simulation results are compared with the test results to verify the accuracy of FLSD as the fracture criterion.

## 2. The Transformation Theory of Stress-Strain

### 2.1. M-K Theoretical Model

The M-K theory proposed by Marciniak Z and Kuczyński K [13] is the most widely used theoretical model for predicting the limit of sheet forming in engineering in recent years.

**Figure 1:**
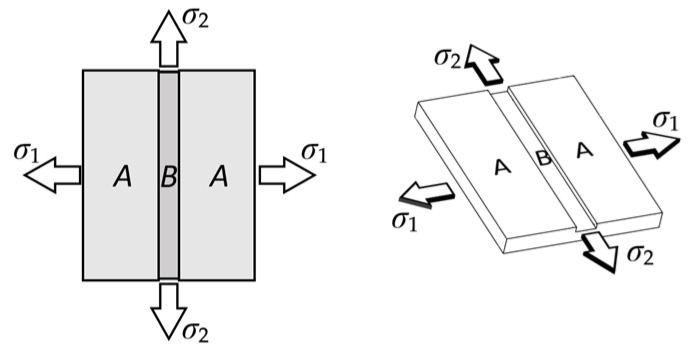
Model of the M-K theory.

Hussein T et al. [14] confirmed that for DP steels, The M-K approach shows the better capability to predict the formability by various loading conditions. In this paper, M-K theory is selected to describe the fracture behavior caused by sheet thickness inhomogeneity. M-K theoretical model is shown in the Figure 1. The contents of M-K theory are as following:

The M-K theory considers the thickness of the sheet metal to be inhomogeneous and that this inhomogeneity is amplified during stretching until fracture. The theory uses a model of a sheet with a groove to carry out the study of a sheet subjected to biaxial stretching. Where zone A is the uniform deformation zone, zone B is the groove zone, and $\sigma_{1}$, $\sigma_{2}$ are the maximum and minimum principal stresses in Figure 1.

This theory was based on some assumptions:

(1) Plastic deformation of the sheet due to biaxial tensile stress, but the volume remains unchanged.

(1) $$d\varepsilon_{1}+d\varepsilon_{2}+d\varepsilon_{3}=0$$

where $d\varepsilon_{1}$, $d\varepsilon_{2}$, $d\varepsilon_{3}$ are the plastic strains of the sheet in three directions.

(2) The second principal strain increment in zone A and zone B are equal.

(2) $$d\varepsilon_{2A}=d\varepsilon_{2B}=d\varepsilon_{2}$$

where $d\varepsilon_{2A}$, $d\varepsilon_{2B}$ are second principal strain increment in zone A and zone B.

(3) The forces in zone A and zone B in the 1st principal direction are in balance

(3) $$\sigma_{1A}t_{A}=\sigma_{1B}t_{B}$$

where $\sigma_{1A}$, $\sigma_{1B}$ are the first principal stresses of zone A and zone B; $t_{A}$, $t_{B}$ are the thickness of zone A and zone B.

(4) When simple loading is applied to Zone A, each principal stress and principal strain increase in proportion, and the ratio is constant:

(4) $$\{\begin{array}{c} \frac{d\sigma_{1A}}{\sigma_{1A}}=\frac{d\sigma_{2A}}{\sigma_{2A}}=\frac{d\sigma_{3A}}{\sigma_{3A}}=\frac{d\sigma_{tA}}{\sigma_{tA}}\\ \frac{d\varepsilon_{1A}}{\varepsilon_{1A}}=\frac{d\varepsilon_{2A}}{\varepsilon_{2A}}=\frac{d\varepsilon_{3A}}{\varepsilon_{3A}}=\frac{d\varepsilon_{tA}}{\varepsilon_{tA}} \end{array}$$

where $d\sigma_{x}$, $d\varepsilon_{x}$ are the incremental principal stress and incremental principal stain, $\sigma_{x}$, $\varepsilon_{x}$ are the principal stress and principal stain.

Combining M-K theory with material constitutive model and corresponding yield criterion, the maximum and minimum limit principal strains $\varepsilon_{1}$ and $\varepsilon_{2}$ can be obtained by iterative calculation, and the sheet FLD can be obtained.

### 2.2. Obtaining Stress State

The limit stress value of metal sheet is difficult to measure in the production. Based on the relationship between stress and strain, the transition from FLD to FLSD can be realized. Panich [15] compared the FLSD of 980 steel calculated from different yield criteria and found that the FLSD data obtained differed significantly according to the different yield criteria. Although the FLSD derived from different theories is not unique, the established FLSD failure criterion will be close to the original FLD results, as long as the same plasticity theory and yield criteria as the FLD calculation model are used in the numerical simulation.

The stress-strain equations in this paper are in the plane-stress ($\sigma_{3}=0$) conditions, $\sigma_{1}$, $\sigma_{2}$ are the maximum and minimum principal stresses.

According to the literature [16,17], the transformation relationship from strain state to stress state is shown in the Equations (5) and (6):

(5) $$\sigma_{1}=\frac{\bar{\sigma }\left[\bar{\varepsilon }\left(\varepsilon_{1i},\varepsilon_{2i}\right)+\bar{\varepsilon }\left(\varepsilon_{1j}-\varepsilon_{1i},\varepsilon_{2j}-\varepsilon_{2i}\right)\right]}{\xi \left\{\alpha \left[\left(\varepsilon_{2j}-\varepsilon_{2i}\right)/\left(\varepsilon_{1j}-\varepsilon_{1i}\right)\right]\right\}}$$

(6) $$\sigma_{2}=\alpha \left(\frac{\varepsilon_{2j}-\varepsilon_{2i}}{\varepsilon_{1j}-\varepsilon_{1i}}\right)\sigma_{1}$$

where $\varepsilon_{1i}$ and $\varepsilon_{2i}$ are the pre strains in two directions, $\varepsilon_{1j}$ and $\varepsilon_{2j}$ are the final strains in two directions, and $\bar{\sigma }\left(\bar{\varepsilon }\right)$ is the hardening function, $\bar{\varepsilon }(\varepsilon )$ is effective strain function, $\alpha \left(\frac{\varepsilon_{2}}{\varepsilon_{1}}\right)$ is the transformation function from $\frac{\varepsilon_{2}}{\varepsilon_{1}}$ to $\frac{\sigma_{2}}{\sigma_{1}}$, ξ(α) is a function of material parameters.

In this paper, the Swift hardening law Equation (7) is used to fit the stress-strain curve of DP steel with uniform elongation.

(7) $$\sigma =K{\left(\varepsilon_{0}+{\bar{\varepsilon }}_{p}\right)}^{n}$$

where σ is stress, *K* is the strength coefficient, *n* is the hardening exponent, $\varepsilon_{0}$ is prestrain, ${\bar{\varepsilon }}_{p}$ is plastic strain.

Based on the non preload test conducted in this study, there is no pre strain. Equations (5) and (6) are transformed into Equations (8) and (9).

(8) $$\sigma_{1}=\frac{\bar{\sigma }\left[\bar{\varepsilon }\left(\varepsilon_{1},\;\varepsilon_{2}\right)\right]}{\xi \left\{\alpha \left[\left(\varepsilon_{2}/\varepsilon_{1}\right)\right]\right\}}$$

(9) $$\sigma_{2}=\alpha \left(\frac{\varepsilon_{2}}{\varepsilon_{1}}\right)\sigma_{1}$$

By different yield criteria, the FLSD obtained is also different. In this paper, based on the Hill’48 yield criteria selected and the Material properties of the four kinds of sheets, the FLSD data are converted. The equivalent stress $\bar{\sigma }$ is solved by the function of the stress $\sigma_{1}$ and $\sigma_{2}$.

(10) $$\bar{\sigma }=\sqrt{\sigma_{1}^{2}+\sigma_{2}^{2}-\frac{2r}{1+r}\sigma_{1}\sigma_{2}}$$

The equivalent strain $\bar{\varepsilon }$ is

(11) $$\bar{\varepsilon }=\frac{1+r}{\sqrt{1+2r}}\sqrt{\varepsilon_{1}^{2}+\varepsilon_{2}^{2}+\frac{2r}{1+r}\varepsilon_{1}\varepsilon_{2}}$$

(12) $$\alpha =\frac{\sigma_{2}}{\sigma_{1}},\;\beta =\frac{\varepsilon_{2}}{\varepsilon_{1}}$$

The proportionality coefficient of equivalent strain to maximum principal stress is

(13) $$\xi =\sqrt{1+\alpha^{2}-\frac{2r}{1+r}\alpha }$$

The relationship between α and β can be defined by

(14) $$\alpha =\frac{\left(1+r\right)\beta +r}{1+r+r\beta }$$

The plastic strain ratio *r*, is the ratio of the true width strain to the true thickness strain at a particular value of length strain.

(15) $$r=\frac{\varepsilon_{b}}{\varepsilon_{t}}$$

where $\varepsilon_{t}$ is the true thickness strain, $\varepsilon_{b}$ is the true width strain.

The *r*-value is provided by the cooperating company, as shown in Table 1.

**Figure 2:**
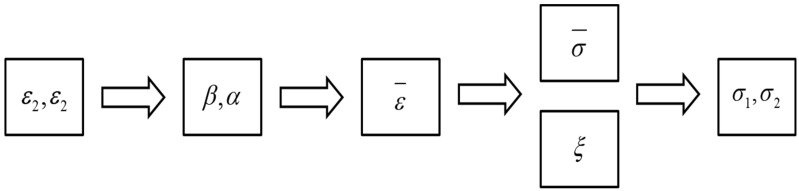
The conversion process of FLSD.

The stress-strain transformation relationship can be solved by combining the above functions. The schematic diagram of the transformation process is shown in Figure 2.

## 3. Experimental Procedure

### 3.1. Experimental Design

The material used in this paper was DP590, DP780, DP980, and DP1180 sheets with a nominal thickness of 1 mm. The microstructure of DP steel mainly consists of two phases: ferrite and martensite. The composition of these four steels was provided by the producer, Shanghai Baosteel Group, and is shown in Table 2. The mechanical properties of the four steels were obtained after metal tensile test, as shown in Table 3, where E is the modulus of elasticity, *ν* is Poisson’s ratio, *K* is strength coefficient and *n* is the hardening exponent.

**Figure 3:**
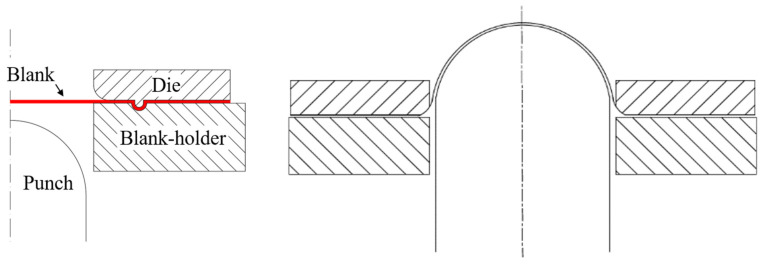
Schematics of the Nakazima test.

Plotting FLD requires obtaining the maximum and minimum principal strain data of the sheet, which can be obtained by Nakazima dome tests on specimens of different sizes [18]. In this paper, experiments are designed with reference to GB/T 15825.8-2008, Sheet metal formability and test methods—Part 8: Guidelines for the determination of forming-limit diagrams. The Nakazima test in this paper was carried out using a YZ32-160S hydraulic press by Zibo Aoheng Hydraulic Machinery Co. (Zibo, China). The Stamping test machine is mainly composed of blank, punch, die and blank holder. The structural principle is shown in Figure 3. During the Nakazima dome test, the blank holder moves downwards to provide sufficient pressure to secure the sheet, and then the punch moves upwards to cause deformation of the sheet until the sheet fractures.

**Figure 4:**
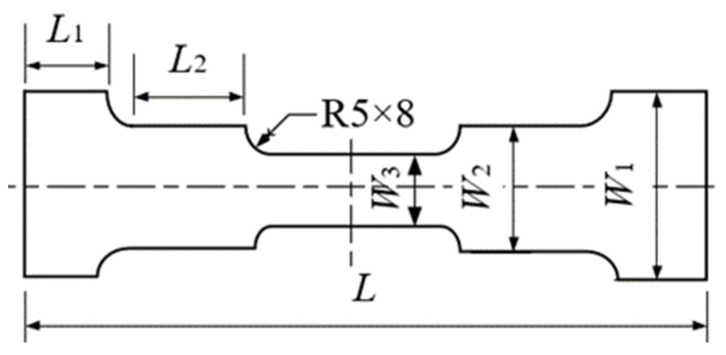
Dimensional drawing of the specimen.

In order to obtain FLD data of DP steel of different grades, three groups of specimens with full length of 200 mm and width varies were designed (Figure 4). The specific dimensions of specimens are shown in Table 4.

**Figure 5:**
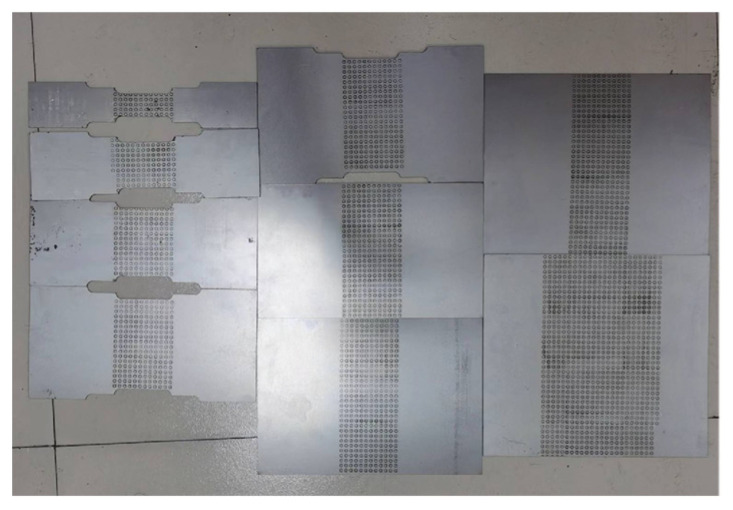
Multiple sizes of specimens.

**Figure 6:**
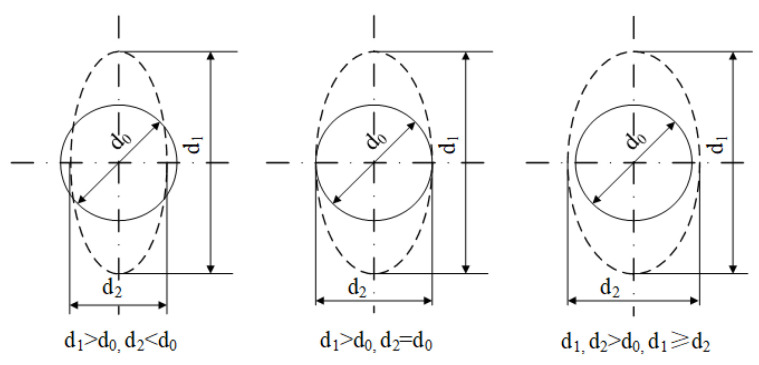
Mesh distortion circle.

Before the Nakazima test, in order to measure the strain of specimens, an electronic marking machine was used to etch an array composed of 1 mm circular grids on each specimen through electrochemical methods (Figure 5). The maximum and minimum principal strains were calculated from the deformation variables of the grid before and after the test. Assume that the circular grid is elliptical after distortion, and the maximum axis is recorded as d_1_ and the minimum axis as d_2_, and the d_1_ and d_2_ are approximately regarded as two principal strain directions on a point in the surface of the specimen sheet. The diagram of grid circle distortion is shown in Figure 6. After the test, the process stress and strain data and the real stress and strain data are calculated according to Equations (16) and (17).

(16) $$\left\{ \begin{array}{l} e_{1}=\frac{d_{1}-d_{0}}{d_{0}}\times 100\\ e_{2}=\frac{d_{2}-d_{0}}{d_{0}}\times 100 \end{array}\right.$$

(17) $$\left\{ \begin{array}{l} \varepsilon_{1}=\ln\frac{d_{1}}{d_{0}}=\ln(1+e_{1})\\ \varepsilon_{2}=\ln\frac{d_{2}}{d_{0}}=\ln(1+e_{2}) \end{array}\right.$$

### 3.2. Experimental Results and Discussion

**Figure 7:**
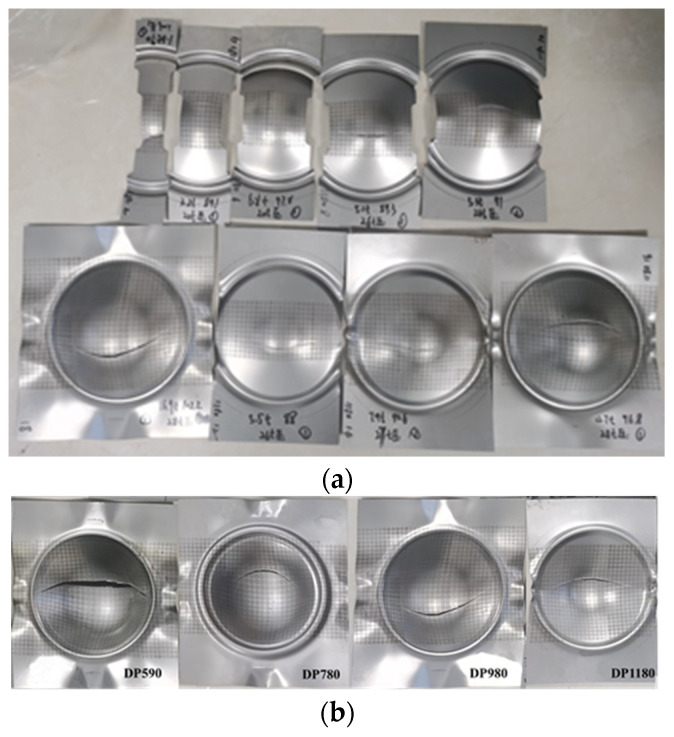
Fracture specimens of Nakazima test. (**a**) Specimens of different sizes; (**b**) Specimens of different materials.

Specimens after the Nakazima test are shown in Figure 7. By observing the fracture specimens in Figure 7, it is found that the places where the fracture surfaces of specimens of different sizes occur are roughly the same. As the tensile and yield strengths of the steel increase, greater blank holder force is required to compress the sheets. The blank holder force increases from about 100 kN for DP590 to about 300 kN for DP1180, while reducing the forming depth and causing earlier failure. Therefore, steel sheet with high tensile strength and yield strength is difficult to be stamped and forming. The fracture surfaces of specimens of most sizes produce arc fracture tracks near the center of the specimen.

**Figure 8:**
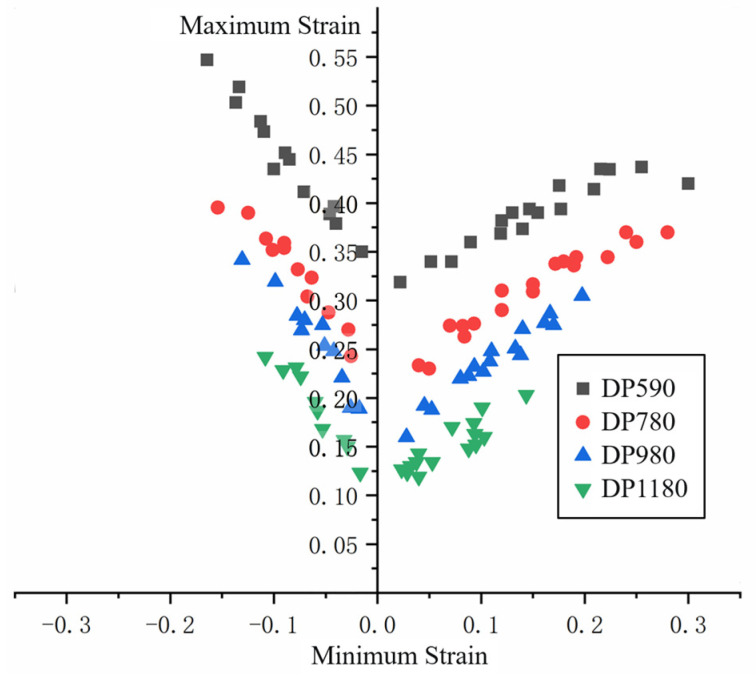
Nakazima test data.

The grid distortion of failure critical area is measured and the data are processed to obtain the maximum and minimum principal strains of each grid. Limit strain data after processing is shown in Figure 8. It is found from the limit strain data of nakazima test that the value of the limit principal strain of sheet forming increases with the increase of the sheet strength, and the value range of the minimum principal strain also expands with the increase of the sheet strength. The trend of the FLC on the left and right sides of the four DP steel is the same, showing a decreasing trend in the left branch as a whole, and an increasing trend in the right branch.

### 3.3. FLD of DP Steel Sheets

Strain data of the FLD from the Nakazima test are not enough to draw an accurate FLC. In order to obtain more strain data, the finite element model of Nakazima test was established in Abaqus. The fracture failure maximum principal strain of some specimens is obtained through simulation to enrich the test data. The accuracy of the simulation model is verified by comparing with fracture results of the Nakazima test.

**Figure 9:**
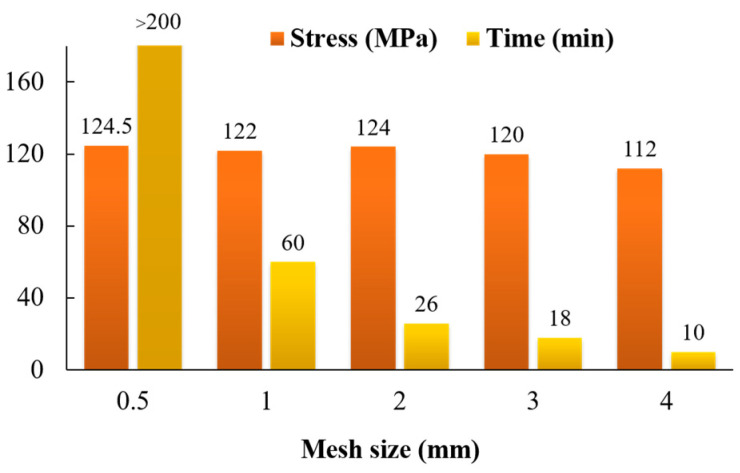
Mesh size analysis results.

Similar to actual experiments, the simulation model is also composed of blank, punch, die and blank holder. The blank is a deformable shell, and other parts are set as rigids. The thickness of blank is set to 1 mm. After mesh size analysis, 2 mm size mesh can save the calculation time and ensure the accuracy of the calculation, the analysis result is shown in the Figure 9. The mesh size of the sheet is 2.0 × 2.0 mm and mass scaling factor was set to 20 so as to reduce the total simulation time. The element type of the blank is S4R.

The simulation uses a user subroutine of Abaqus, VUMAT. VUMAT is used to define the constitutive model of materials which is written in fortran. When the subroutine is called, it is provided with state at the start of the increment (stress, solution-dependent state variables) of blocks of material points. The simulation program applies the Hill (1948) orthotropic yield criterion and Swift hardening law Equation (7) for the calculation.

The Hill (1948) orthotropic yield condition reads

(18) $$\bar{\sigma }=\sqrt{F{\left(\sigma_{22}-\sigma_{33}\right)}^{2}+G{\left(\sigma_{33}-\sigma_{11}\right)}^{2}+H{\left(\sigma_{33}-\sigma_{11}\right)}^{2}+2L\sigma_{23}^{2}+2M\sigma_{31}^{2}+2N\sigma_{12}^{2}}$$

where $\bar{\sigma }$ is the Hill (1948) equivalent stress, $\sigma_{x}$ represents the component of the Cauchy stress tensor, the six constants, *F*, *G*, *H*, *L*, *M*, *N* are anisotropic parameters.

Anisotropy parameters of DP steel sheet in Hill’48 anisotropy yield criterion selected in this paper are calculated by the plastic strain ratio *r* in the previous paper, and their relationship is shown in Equation (19). *L*, *M*, and *N* are difficult to distinguish, and it is generally considered that *L* = *M* = *N*. Anisotropic parameters of these four steels is shown in Table 5.

(19) $$F=\frac{r_{0}}{r_{90}\left(r_{0}+1\right)},\;\;\;\;\;G=\frac{1}{r_{0}+1},\;\;\;\;\;H=\frac{r_{0}}{r_{0}+1},\;\;\;\;\;N=\frac{\left(r_{0}+r_{90}\right)\left(1+2r_{45}\right)}{2r_{90}(1+r_{0})}$$

**Figure 10:**
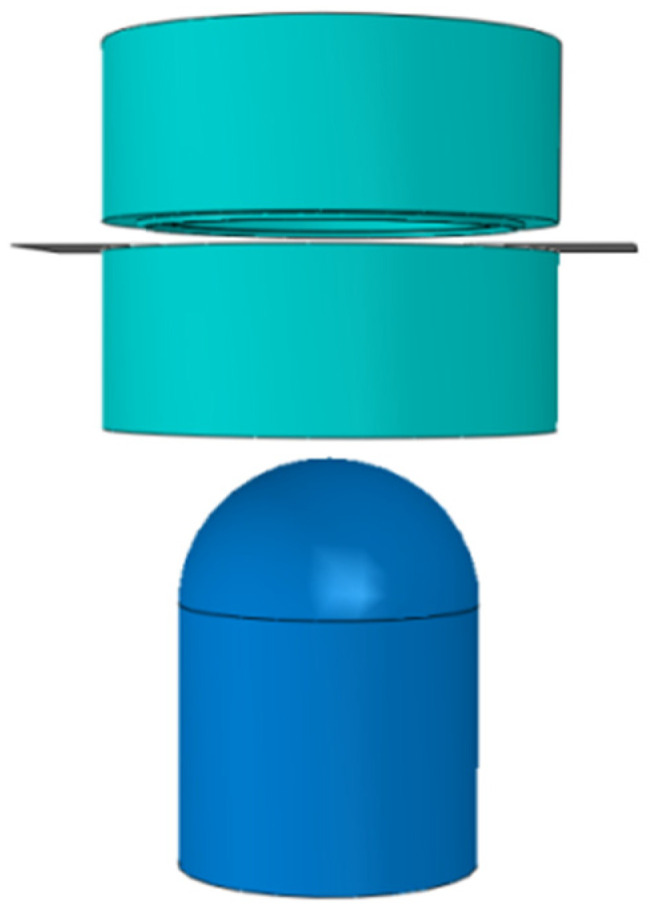
Nakazima test simulation model diagram.

Establishing contact and friction on the surfaces of the rigids and the deformable shell, and the friction coefficient of the whole model is set to 0.15. Limit all degrees of freedom of the die during the simulation. The first step compresses the blank downwards with the blank holder, and the second step deforms the blank with the punch upwards. The finite element model of the Nakazima test is illustrated in Figure 10.

**Figure 11:**
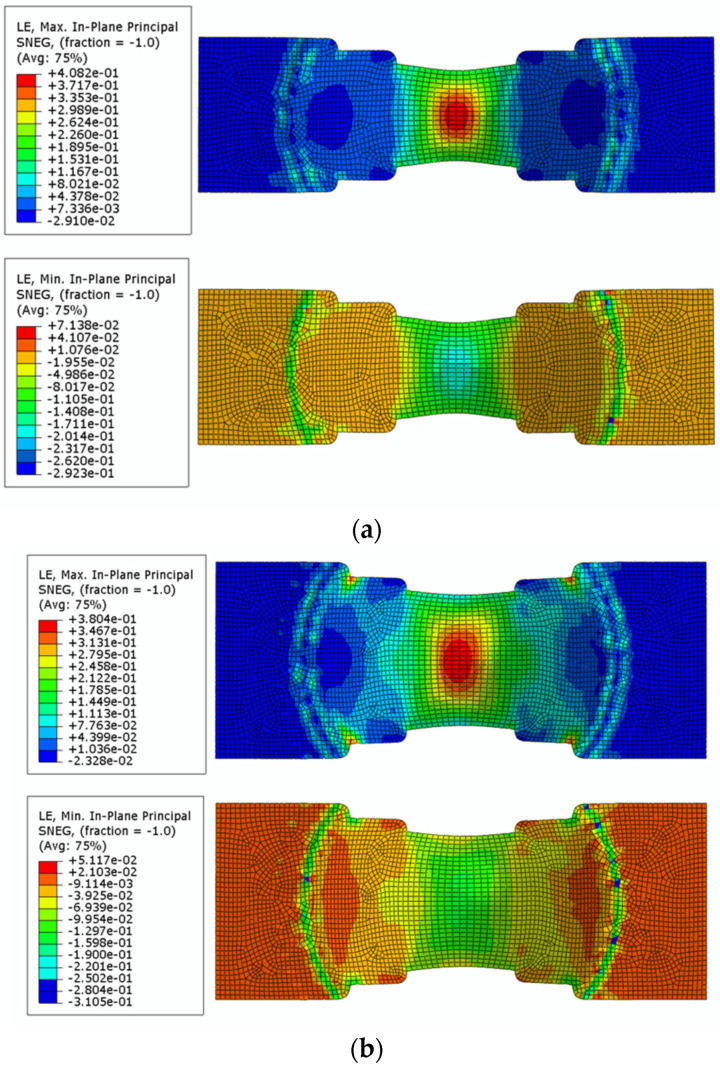

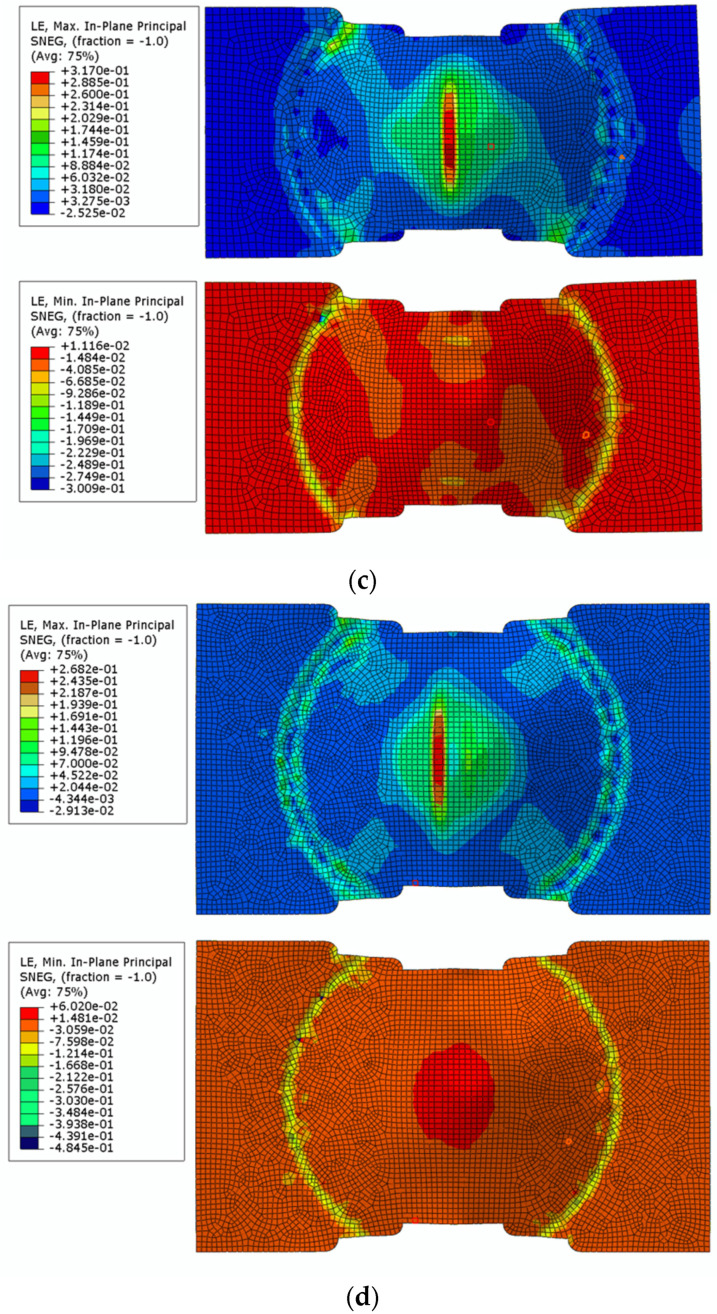
Simulated strain nephogram of Nakazima test of some sheets. (**a**) W1 = 40 mm; (**b**) W1 = 60 mm; (**c**) W1 = 80 mm; (**d**) W1 = 100 mm.

The bulge fracture simulation was carried out for DP590, DP780, DP980 and DP1180 respectively, and the strain nephograms at the fracture time were obtained. The simulated strain nephograms of some sheets are shown in Figure 11. At the same time, the maximum and minimum limit principal strain data of the bulge specimens of different sizes are recorded and sorted out.

**Figure 12:**
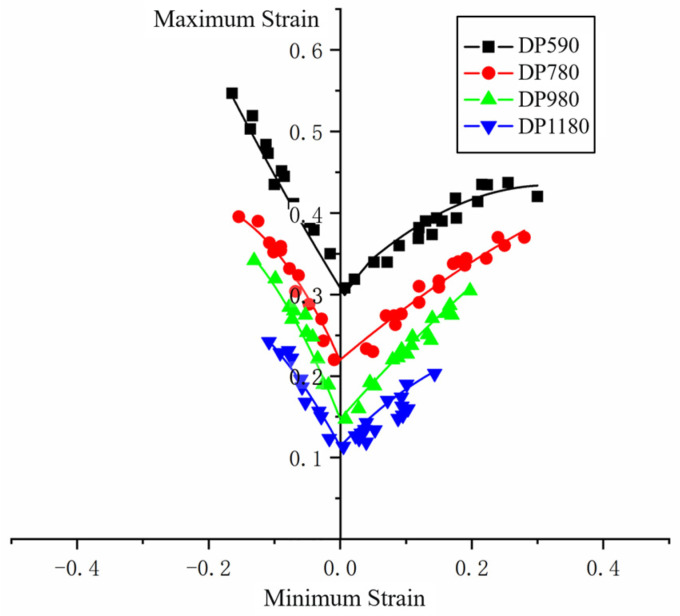
Forming limit diagram after fitting.

After obtaining the limit strain data and FLD_0_ points of each sheet, binary linear fitting is carried out. The expression after fitting is shown in Equation (20), and the goodness of fit is 0.968. Combine the test data points with the simulation data points to form FLD, as shown in Figure 12. The lowest point of the diagram is obtained based on Equation (20). Curves on both sides of the lowest point are fitted based on Equation (21).

(20) $$FLD_{0}=1.656*n+0.032*t-0.025$$

where *n* is the hardening exponent, *t* representing the thickness of the steel sheet (only steel sheets with thickness less than 3 mm are considered in this paper).

(21) $$\varepsilon_{1}=FLD_{0}+\varepsilon_{2}(nd_{1}*\varepsilon_{2}+d_{2})$$

where *n* is the hardening exponent, *d*_1_ and *d*_2_ are parameters to be fitted. Their fitted values are shown in Table 6.

### 3.4. Forming Limit Stress Diagram

**Figure 13:**
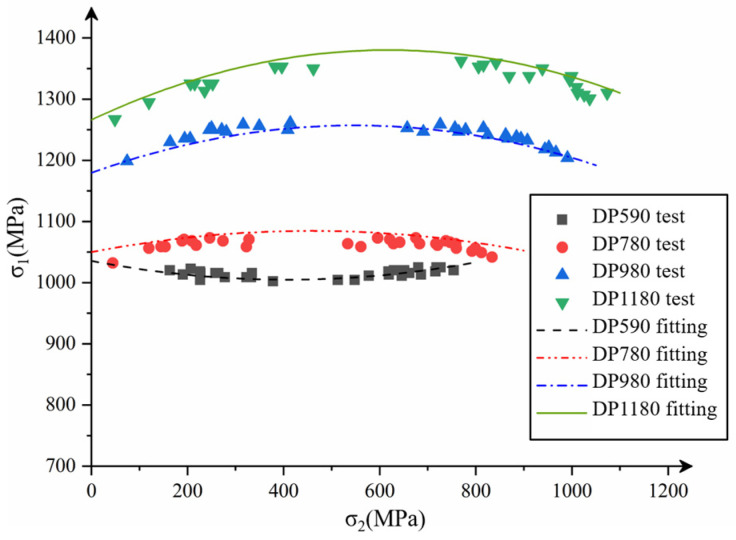
FLSD of four dual-phase steel materials.

The limit stress data of DP590, DP780, DP980 and DP1180 are determined by combining the conversion function with the performance parameters of the four kinds of sheets. To fit the FLSD formula, the FLSD fitting curves of DP590, DP780, DP980 and DP1180 are established, and the limit stress data points are processed. Observe the curves in the Figure 13, the trend is relatively gentle compared with FLD. The limit principal stress ranking between each type of DP steel is consistent with the strength ranking of the steel. The curves are obtained by multiple linear regression fitting in combination with the performance parameters of the sheets, the coefficient of the quadratic term of curves is related to the hardening exponent *n.* as shown in Equation (22). The expression established after fitting is as follows:

(22) $$\sigma_{1}=(0.02n^{2}-7\times 10^{-4}n-3.3\times 10^{-4})\sigma_{2}^{2}+\left(0.633-4.38n\right)\sigma_{2}+0.72K+327$$

where $\sigma_{1}$, $\sigma_{2}$ are the maximum and minimum limit principal stresses, *K* is the strength coefficient, and *n* is the hardening exponent.

Due to fluctuations in the stress data obtained through experiments, there are inevitable errors in the fitting formula. The RMSE (root mean squared error) is a standard for intuitively testing the accuracy of regression equations.

(23) $$RMSE=\sqrt{\frac{1}{N}\sum_{i=1}^{n}{\left(Y_{i}-f\left(x_{i}\right)\right)}^{2}}$$

where *n* is number of items, $Y_{i}$ is original or observed value, $f\left(x_{i}\right)$ is value from fitting formula.

The RMSE calculated from the FLSD of DP590, DP780, DP980 and DP1180 are 6.64, 10.50, 11.70, 12.91. This means that the fitting formula in this study needs to consider the existence of small errors when applied.

According to the expression observation curve and the strain hardening function, when solving the equivalent stress $\bar{\sigma }$, its value is exponentially related to the strength coefficient *K* and hardening exponent *n*. Therefore, the limit stress value in the FLSD curve increases with the increase of *K* and *n*. The *K* and the strength of the sheet have the same trend of change in the performance, while the *n* decreases with the increase of the strength of the sheet. The function shows that the k-value determines the value of the maximum principal stress, while the n-value affects the curvature of the curve. It can be seen from the Figure 13 that the limit stress value of DP1180 sheet is greater than that of DP590, so the limit stress value in the sheet generally increases with the increase of sheet strength, and the influence of strength coefficient *K* on FLSD is greater than that of hardening exponent *n*.

## 4. Application

### 4.1. Nakazima Test Simulation

The FLSD fitting formulas obtained in the previous chapter can guide sheet metal forming by computer simulation. The settings of the simulation process are basically the same as above. FLSD can be conveniently applied to form a simulation as a fracture criterion by using VUMAT subprogram in Abaqus. The simulation model uses shell elements, which define two principal stresses in the plane direction and the principal stresses in the normal direction is zero. Call the two principal stresses of the shell element through the vumat subroutine, and divide the two principal stresses into the maximum principal stress and the minimum principal stress based on their magnitude relationship. Once the numerical relationship between the maximum principal stress and the minimum principal stress of a element exceeds the limit value determined by the FLSD function, the element will be deleted to simulate failure.

**Figure 14:**
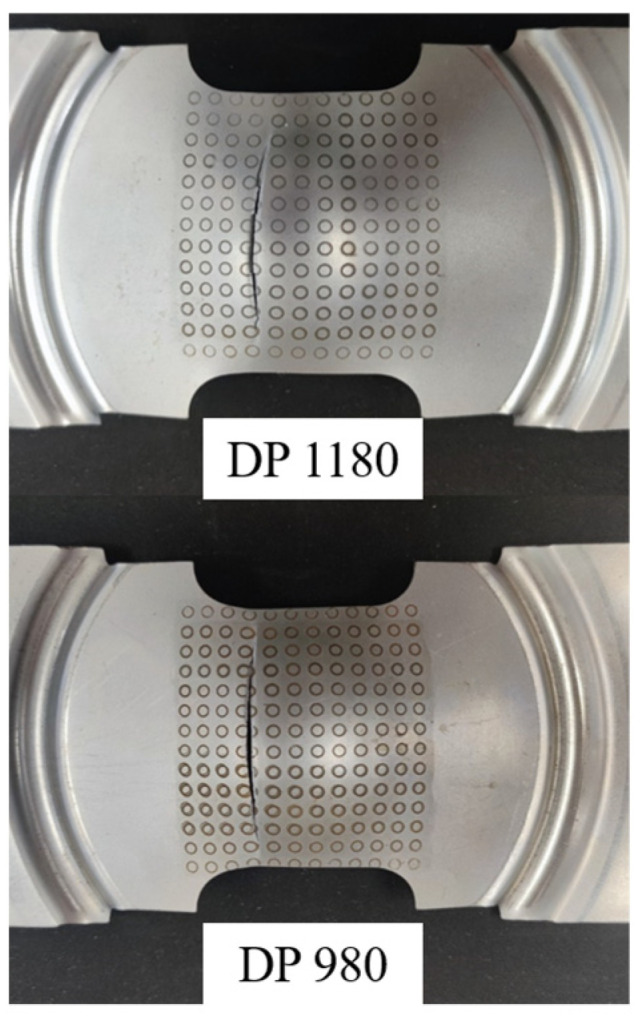
Nakazima test of specimens with a width of 60.

**Figure 15:**
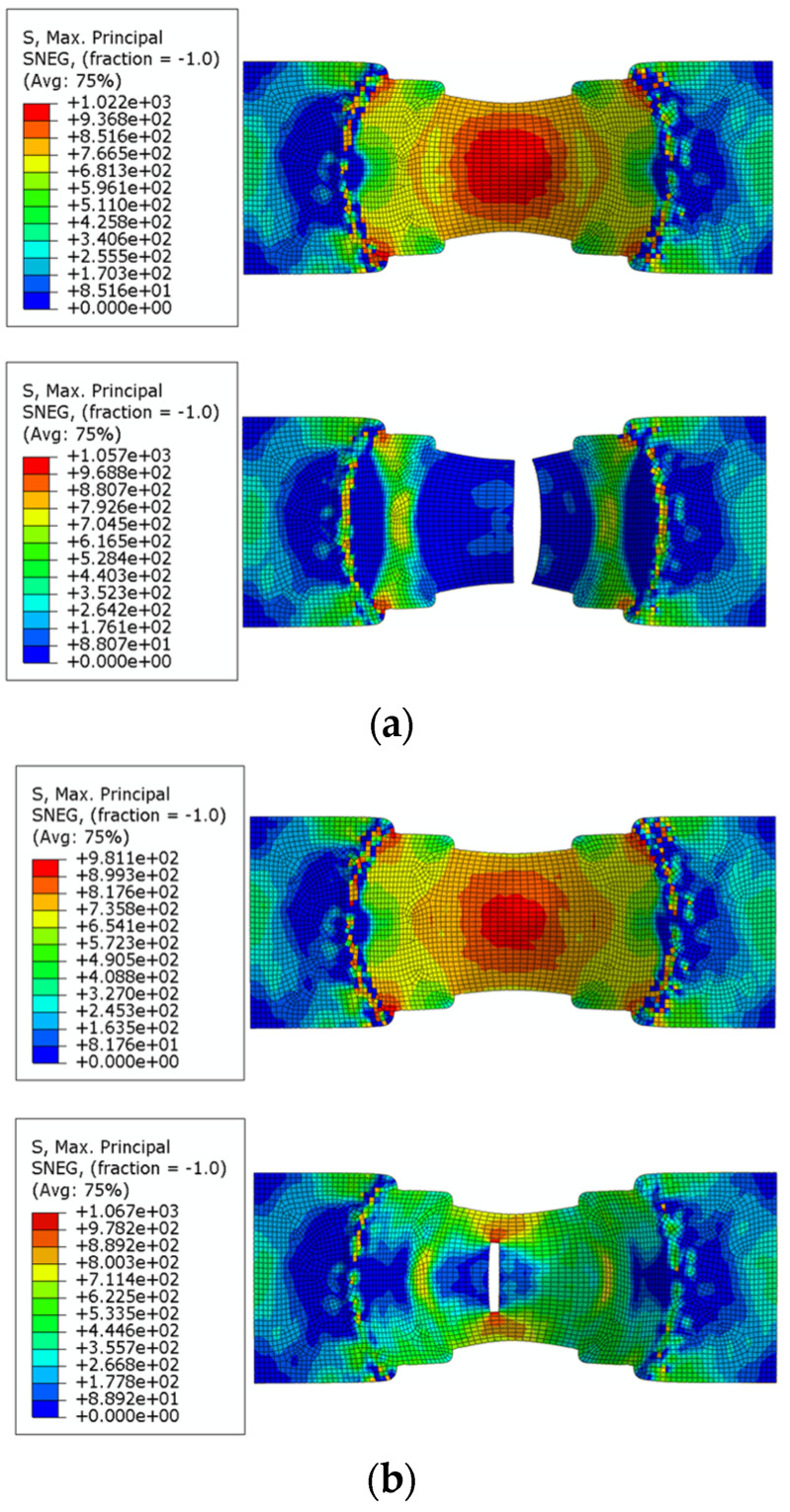

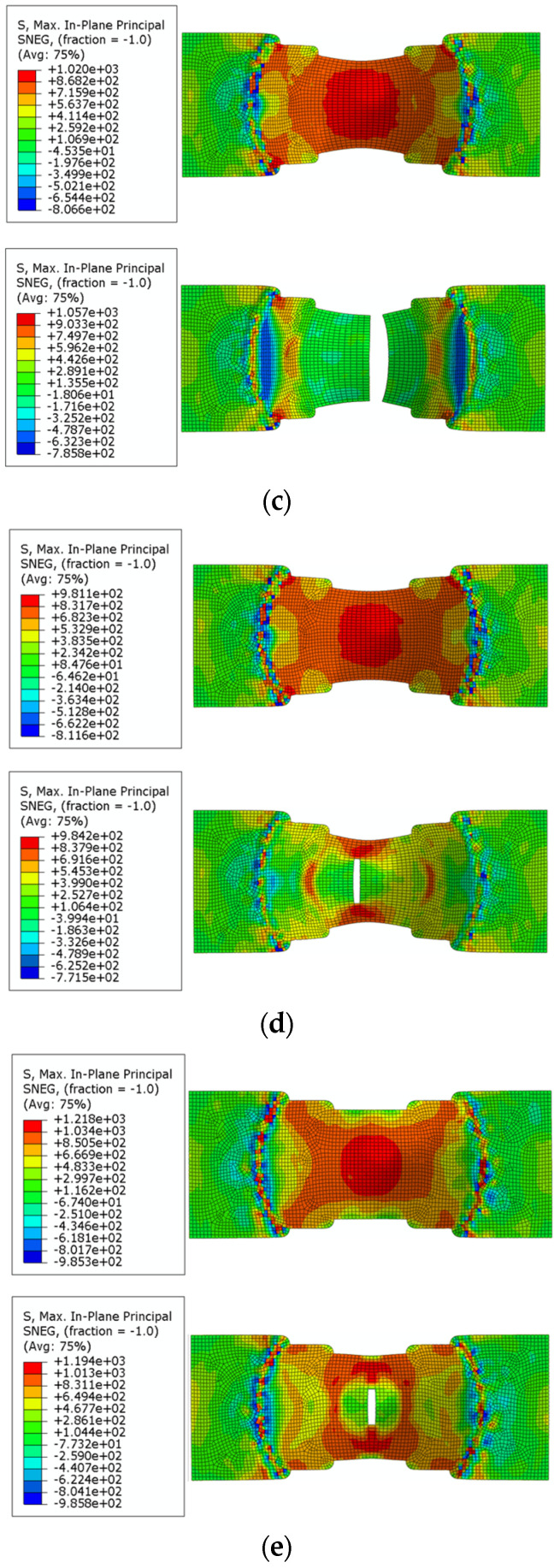

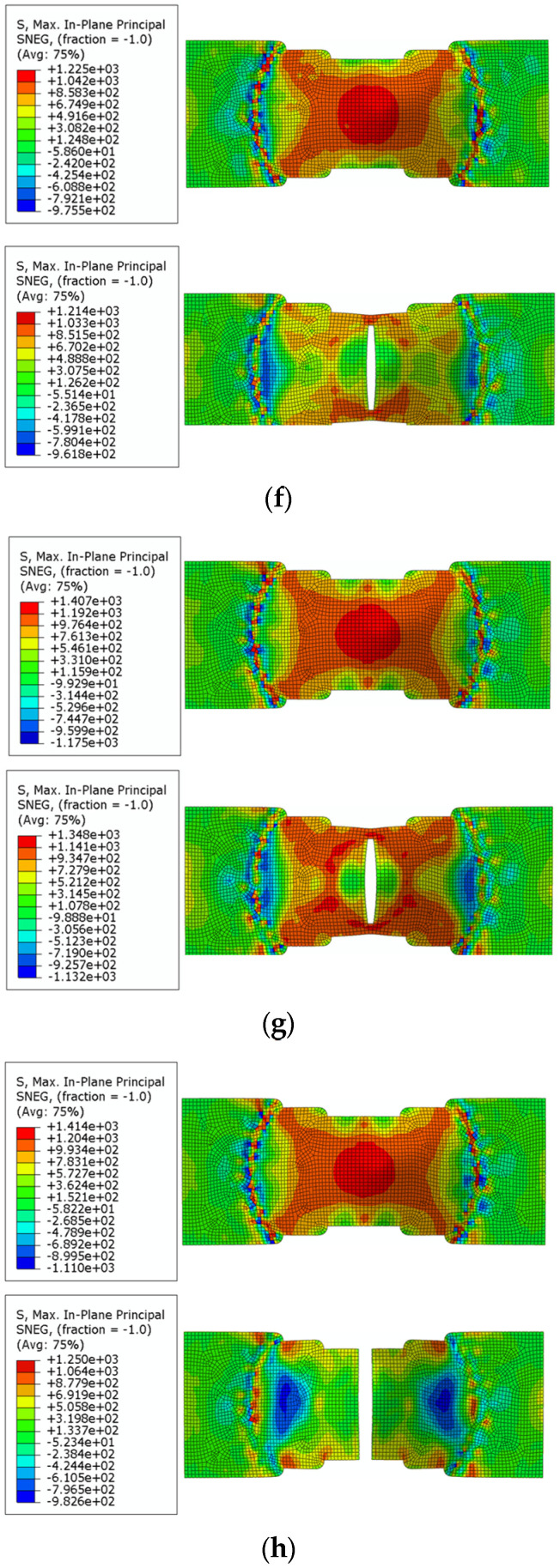
Comparison of FLSD and FLD simulation experiments, (**a**) FLD simulation test of DP590; (**b**) FLSD simulation test of DP590; (**c**) FLD simulation test of DP780; (**d**) FLSD simulation test of DP780; (**e**) FLD simulation test of DP980; (**f**) FLSD simulation test of DP980; (**g**) FLD simulation test of DP1180; (**h**) FLSD simulation test of DP1180.

Taking the bulging test of specimens with a width of 60 as an example, Figure 14 shows the test results and Figure 15 shows the simulation results. The first diagram of each simulation test shows the maximum principal stress distribution at the moment before fracture, while the second shows the principal stress distribution at the moment of fracture. A comparison of test and simulation fracture depth is shown in the Table 7.

Using FLSD as the failure criterion for the simulation model of the Nakazima dome test, the calculation results are basically consistent with the FLD and also consistent with the experimental results, which proves the reliability of FLSD as a fracture criterion.

### 4.2. Box Forming Test

**Figure 16:**
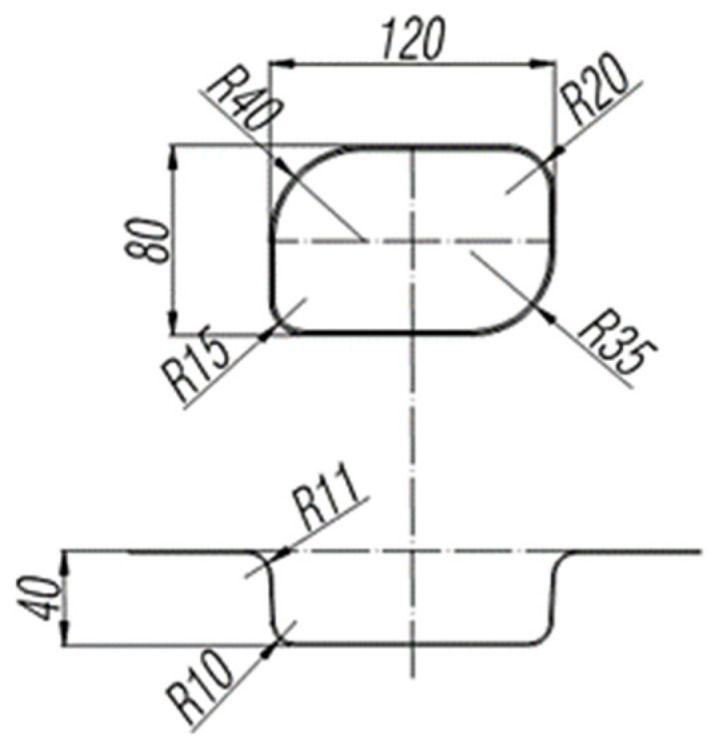
Box mold size.

**Figure 17:**
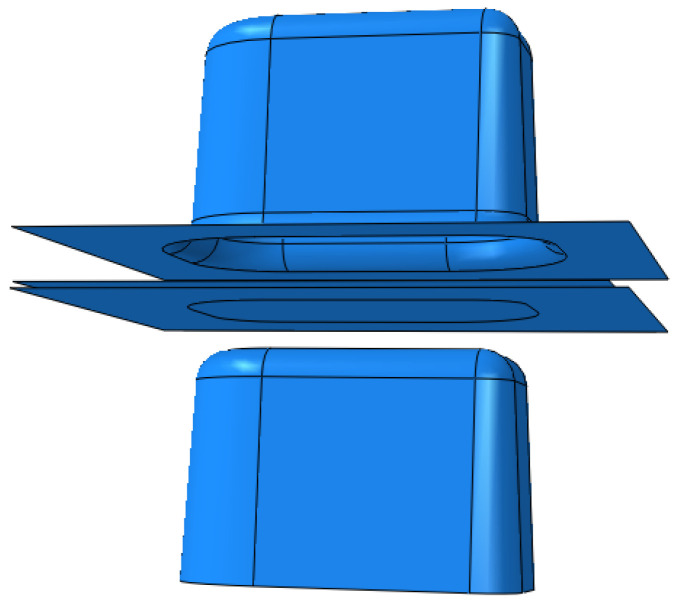
Box forming model.

In order to verify the fitted forming limit curve, the box forming test was carried out by YZ32-160S hydraulic press. The test equipment was roughly the same as the bulging tests. Punch and blank holder are replaced with box die. In order to study the effect of different fillet on the forming effect, a box die with four sizes of fillet was experimentally designed. The size of box die with unequal fillet is shown in Figure 16. The rectangular sheet of DP590, DP780, DP980, and DP1180 were tested by the press with the size of 190 mm in length and 180 mm in width. A simulation model was designed based on the mold size the simulation model is shown in Figure 17. Simulating the forming process of the box forming experiment, and the limit stress value is obtained. FLSD damage data is imported into Abaqus, and the accuracy of FLSD data of each sheet is verified by outputting the maximum principal stress through damage evolution settings.

**Figure 18:**
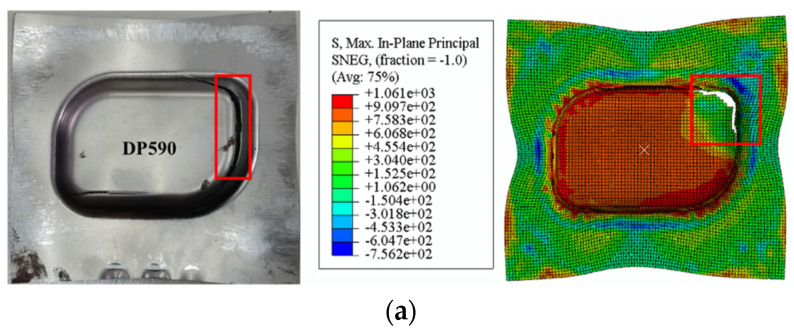

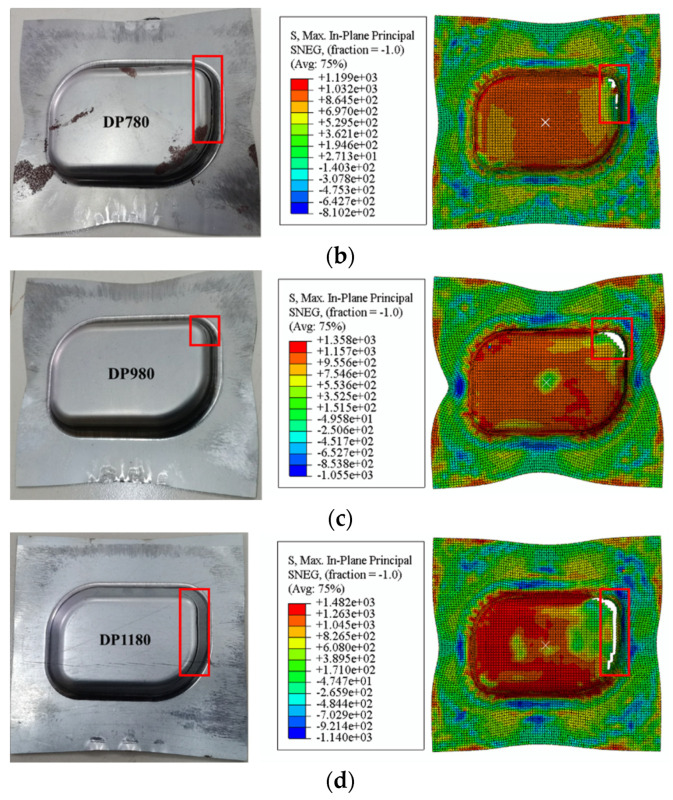
Principal stress distribution at the moment of fracture (**a**) DP590; (**b**) DP780; (**c**) DP980; (**d**) DP1180.

The test and simulation results of the box forming and the simulated principal stress values are shown in Figure 18. By observing the FLSD simulation results of the four types of sheet DP590, DP780, DP980 and DP1180, and comparing maximum and minimum principal stress values obtained from the stamping forming test and simulation of each type of sheet box, it is found that the maximum principal stress gap of the four types of sheet is large and the minimum principal stress gap is small. Maximum principal stress of DP590 is kept around 1000 MPa; the maximum principal stress simulation value of DP780 is about 1100 MPa; the maximum principal stress of DP980 fluctuates around at 1200 MPa; the maximum principal stress of DP1180 reaches 1300 MPa. The maximum principal stress and the minimum principal stress values of the four kinds of DP steel sheet show an increasing trend, but the growth range of the minimum principal stress is small with the increase of the sheet strength. The comparison of box forming test and simulated stamping depth is shown in the Table 8.

Through comparative test and simulation, the stamping depth value and test error of the simulation model are less than 10% when the blank holder force value is the same, which effectively shows that the FLSD values of sheets are consistent with the established FLSD data, and proves the accuracy of the established FLSD.

## 5. Conclusions

In this paper, the Nakazima test of DP590, DP780, DP980 and DP1180 is designed, and the required forming limit data points of four materials are obtained. The numerical simulation of Nakazima test is carried out to improve the accuracy of FLD fitting. Finally, the FLD and its unified expression of four kinds of dual phase steel sheets are established. Based on the stress-strain relationship of M-K theory, the conversion between FLD and FLSD is realized, and the FLSD of four kinds of dual phase steel sheets is established. By studying the correlation with the sheet property parameters, the unified FLSD expression of four kinds of dual phase steel sheets is established. The main conclusions of this research are:

(1) The limit stress data of the four kinds of DP steel (DP590, DP780, DP980, DP1180) were obtained and FLSD was plotted through the Nakazima dome test and simulation. The differences in limit stresses of the four steels and the effect of such differences on forming were compared. It was verified by numerical simulation that the FLSD obtained in this paper is within 10% error from experiment and can be used as a fracture criterion to accurately simulate the fracture of DP steel sheets.
(2) The influence strength coefficient *K* and hardening exponent *n* of DP steel of FLSD function is analyzed, and these parameters are introduced into the FLSD function. The *K* and the strength of the sheet have the same trend of change in the performance, while the *n* decreases with the increase of the strength of the sheet. The function shows that the *k*-value determines the value of the maximum principal stress, while the *n*-value affects the curvature of the curve. A high fitting FLSD calculation method is obtained. The FLSD function proposed in this paper can be extended to the fracture calculation of DP steel with other strength grade, reducing the difficulty of FLSD application.

## Figures and Tables

**Table 1:** R-values for the four DP steels.

Steel	*r*_0_	*r*_45_	*r*_90_	$\bar{r}$
DP590	0.80	0.87	1.01	0.89
DP780	0.79	0.84	0.79	0.82
DP980	0.75	0.80	0.87	0.83
DP1180	0.82	0.95	0.98	0.93

**Table 2:** Sheet element composition and mass fraction%.

Steel	C%	Si%	Mn%	P%	S%	Al%
DP590	0.08	0.46	1.75	0.014	0.004	0.032
DP780	0.1	0.16	2.02	0.008	0.003	0.039
DP980	0.179	1.714	2.25	0.01	0	0.029
DP1180	0.118	0.23	2.48	0.008	0.001	0.031

**Table 3:** Material properties.

Steel	E (MPa)	*ν*	*K* (MPa)	*n*
DP590	201,000	0.28	980	0.179
DP780	215,000	0.29	1000	0.11
DP980	216,000	0.29	1180	0.08
DP1180	218,000	0.29	1300	0.06

**Table 4:** Dimension of Nakazima test specimen.

Length	Specimen								
	1	2	3	4	5	6	7	8	9
L	180	180	180	180	180	180	180	180	180
L1	40	40	40	40	40	-	-	-	-
L2	20	20	20	20	20	-	-	-	-
W1	40	60	80	100	120	120	120	160	180
W2	30	50	70	90	110	120	140	160	180
W3	20	40	60	80	100	120	140	160	180

**Table 5:** Hill’s constants of steel sheets.

Steel	*F*	*G*	*H*	*L*	*M*	*N*
DP590	0.44	0.56	0.45	1.36	1.36	1.36
DP780	0.56	0.56	0.44	1.5	1.5	1.5
DP980	0.49	0.57	0.43	1.38	1.38	1.38
DP1180	0.46	0.55	0.45	1.46	1.46	1.46

**Table 6:** FLD fitting expression parameters.

Steel	*n*	*d*_1_		*d*_2_		R^2^
		Left Branch	Right Branch	Left Branch	Right Branch	
DP590	0.179	4.0118	−6.9519	−1.3138	0.7920	0.960
DP780	0.11	−35.4332	−3.7913	−1.7413	0.6830	0.965
DP980	0.08	−51.1232	−6.5013	−2.0413	0.9198	0.986
DP1180	0.06	−45.2388	−23.2488	−1.4858	0.8418	0.968

**Table 7:** Comparison of test and simulation fracture depth.

Steel	FLSD (mm)	FLD (mm)	Test (mm)
DP590	27.8	27.2	26
DP780	22.5	22.1	21
DP980	16.4	16.1	15
DP1180	14.1	13.6	13

**Table 8:** Comparison of box forming test and simulated stamping depth.

Steel	FLSD (mm)	Test (mm)	Difference
DP590	27.6	29	5%
DP780	25.4	26	2.3%
DP980	21.2	23	7.8%
DP1180	19.4	21	7.6%

## Data Availability

Not applicable.
